# Direct Oral Anticoagulants vs. Warfarin in Latin American Patients With Atrial Fibrillation: Evidence From Four *post-hoc* Analyses of Randomized Clinical Trials

**DOI:** 10.3389/fcvm.2022.841341

**Published:** 2022-03-04

**Authors:** Fuwei Liu, Yunhong Wang, Jun Luo, Lin Huang, Wengen Zhu, Kang Yin, Zhengbiao Xue

**Affiliations:** ^1^Department of Cardiology, The Affiliated Ganzhou Hospital of Nanchang University, Ganzhou, China; ^2^Department of Cardiology, The Second Affiliated Hospital of Nanchang University, Nanchang, China; ^3^Department of Cardiology, The First Affiliated Hospital of Sun Yat-sen University, Guangzhou, China; ^4^Department of Critial Care Medicine, The First Affiliated Hosptial of Gannan Medical University, Ganzhou, China

**Keywords:** atrial fibrillation, direct oral anticoagulants, warfarin, Latin American, meta-analysis

## Abstract

**Background:**

Several studies have investigated the effect of direct oral anticoagulants (DOACs) in Latin American patients with atrial fibrillation (AF), but the results remain controversial. Therefore, we aimed to compare the efficacy and safety of DOACs vs. warfarin in Latin American patients with AF.

**Methods:**

We systematically searched the PubMed and Embase databases until November 2021 for studies that compared the effect of DOACs vs. warfarin in Latin patients with AF. Adjusted hazard ratios (HRs) and 95% CIs were pooled by a random-effects model using an inverse variance method.

**Results:**

Four *post-hoc* analyses of randomized clinical trials (RCTs) involving 42,411 DOACs and 29,270 warfarin users were included. In Latin American patients with AF, for the effectiveness outcomes, the use of DOACs compared with warfarin was significantly associated with decreased risks of stroke or systemic embolism (SSE) (HR = 0.78; 95%CI.64–0.96), stroke (HR = 0.75; 95%CI.57–0.99), hemorrhagic stroke (HR = 0.14; 95%CI.05–0.36), all-cause death (HR = 0.89; 95% CI.80–1.00), but not ischemic stroke and cardiovascular death. For the safety outcomes, compared with warfarin, the use of DOACs was associated with reduced risks of major or non-major clinically relevant (NMCR) bleeding (HR = 0.70; 95% CI.57–0.86), major bleeding (HR = 0.70; 95%CI.53–0.92), intracranial hemorrhage (ICH) (HR = 0.42; 95%CI.24–0.74), or any bleeding (HR = 0.70;95% CI.62–0.78), but not gastrointestinal bleeding. In non-Latin American patients with AF, for the effectiveness outcomes, the use of DOACs compared with warfarin was significantly associated with decreased risks of SSE (HR = 0.87; 95%CI.75–1.00), hemorrhagic stroke (HR = 0.41; 95%CI.28–0.60), cardiovascular death (HR = 0.87; 95% CI.81–0.94), all-cause death (HR = 0.90; 95% CI.85–0.94). Conversely, the risk of myocardial infarction increased (HR = 1.34; 95% CI 1.13–1.60), but not ischemic stroke. For the safety outcomes, compared with warfarin, the use of DOACs was associated with reduced risks of major or NMCR bleeding (HR = 0.75; 95%CI.61–0.92), major bleeding (HR = 0.76; 95%CI.63–0.92), ICH (HR = 0.42; 95%CI.36–0.52), and any bleeding (HR = 0.81; 95% CI.71–0.92), but not gastrointestinal bleeding.

**Conclusion:**

Current pooled data from the four *post-hoc* analyses of RCTs suggested that compared with warfarin, DOACs appeared to have significant reductions in SSE, stroke, hemorrhagic stroke, all-cause death, major or NMCR bleeding, major bleeding, ICH, and any bleeding, but comparable risks of ischemic stroke, cardiovascular death, and gastrointestinal bleeding in Latin American patients with AF. DOACs appeared to have significant reductions in SSE, hemorrhagic stroke, all-cause death, cardiovascular death, major or NMCR bleeding, major bleeding, ICH, and any bleeding, and increased the risk of myocardial infarction, but comparable risks of stroke, ischemic stroke, and gastrointestinal bleeding in non-Latin American patients with AF.

## Introduction

Atrial fibrillation (AF) is the most common arrhythmia in adults. The currently estimated prevalence of AF in adults ranges from 2 to 4%, and a 2.3-fold rise is expected due to the longevity in the general population and the increased screening of patients with previously undiagnosed AF (1). Advanced age is widely regarded as a foremost risk factor, but increasing burden of other comorbidities including hypertension, diabetes mellitus, heart failure, coronary artery disease, chronic kidney disease, obesity, and obstructive sleep apnea also contributes to the higher prevalence of AF. Not only that, many modifiable risk factors are potent contributors to AF development and progression (2). Many cardiovascular and cerebrovascular complications, such as 5-fold rise in stroke and 2 times the risk of mortality, are prevalently screened in patients with AF (3). AF-associated thromboembolic events are lead contributors for the poor prognosis in patients with AF, which involves higher morbidity and mortality (4, 5). Antithrombotic therapy effectively reduces the incidence of embolism in patients with AF. Direct oral anticoagulants (DOACs) have superior effectiveness and safety outcome for the prevention of stroke and thromboembolic events in patients with AF (6). DOACs are recommended as preferred alternatives to warfarin in the American College of Cardiology and/or American Heart Association/Heart Rhythm Society (7) and European Society of Cardiology guidelines (8) due to its superior characteristics in effectiveness, safety, and convenience, especially for elderly patients with acute coronary syndrome or chronic kidney disease (9, 10). Important differences in clinical characteristics, response to treatment, and outcomes of patients with AF distribute to the diverse regions of the world. In Latin America, AF is regarded as a considerable cause of high mortality and disability (11). Although prevalence data is limited, the incidence of AF-related stroke and associated morbidity is increasing in this region (12), and anticoagulation is underused (13). Therefore, patients with AF in Latin America undergo higher risk of death and thromboembolic events due to the aging population and poorly managed risk factors of AF, such as hypertension, diabetes, heart failure, etc. The anticoagulant treatment of patients with AF is particularly significant in Latin America.

Several previously published studies demonstrated that patients with AF in Latin America treated with warfarin had higher adjusted mortality rates and incidence of stroke and/or systemic embolism, intracranial hemorrhage, and life-threatening or fatal bleeding compared with patients with AF in the rest of the world (ROW) (14). Data regarding the effectiveness and safety outcome of anticoagulation regimens in this region is insufficient. Although several new *post-hoc* analyses of randomized clinical trials (RCTs) well-examined the association between regions (Latin America vs. non-Latin America) and effectiveness and safety outcomes, even explored the use of individual DOACs compared with warfarin in Latin American patients, the superiority of DOACs therapy is still controversial. Although, a previous meta-analysis included the *post-hoc* analyses and sub-analyses of DOACs, RCTs identified a non-inferiority of DOACs compared with warfarin in Latin American patients with AF (15). However, the RCTs included in this meta-analysis are outdated. New RCTs have been published in recent years and report more endpoint events and even find different results. Therefore, we aimed to reassess the effectiveness and safety outcomes of DOACs vs. warfarin in Latin American and non-Latin American patients with AF.

## Methods

### Literature Retrieval

The two common databases of PubMed and Embase were systematically searched until November 2021 for the available studies using the following search terms: (1) atrial fibrillation (2) non-vitamin K antagonist oral anticoagulants OR direct oral anticoagulants OR dabigatran OR rivaroxaban OR apixaban OR edoxaban, and (3) vitamin K antagonists OR warfarin. The detailed search strategies are shown in Supplementary Table 1. In this meta-analysis, we included publications in English.

### Inclusion and Exclusion Criteria

We included the *post-hoc* analyses of RCTs focusing on the effectiveness and/or safety of DOACs (dabigatran, rivaroxaban, apixaban, or edoxaban) compared with warfarin in Latin American patients with non-valvular AF. The effectiveness outcomes included stroke or systemic embolism (SSE), stroke, ischemic stroke, hemorrhagic stroke, ischemic stroke, all-cause death, cardiovascular death, and myocardial infarction; whereas the safety outcomes included major bleeding, major or non-major clinically relevant (NMCR) bleeding, intracranial hemorrhage (ICH), gastrointestinal bleeding, and any bleeding. The follow-up time was not restricted. We excluded certain publication types such as reviews, case reports, case series, editorials, letters, and meeting abstracts because they had no sufficient data. Studies with overlapping data were also excluded.

### Study Screenings and Data Extraction

Two authors (FW-L and YH-W) independently did the data extraction. We first screened the titles and abstracts of the searched records to select potential studies, and the full text of which was screened in the subsequent phase. Disagreements were resolved through discussion or consultation with the third researcher (WG-Z). Two authors independently collected the following characteristics: the first author and publication year, location, data source, study design, inclusion period, patient age and sex, type or dose of DOACs, follow-up time, effectiveness and safety outcomes, the sample size, and the number of events in the vitamin K antagonist (VKA) or DOAC groups, and adjusted hazard ratios (HRs) and 95% CIs.

### Quality Assessment

We used the Newcastle-Ottawa Scale (NOS) to perform the quality assessment for the included studies. The NOS tool had three domains, scored a total of 9 points including the selection of cohorts (4 points), the comparability of cohorts (2 points), and the assessment of the outcome (3 points). In this study, we defined studies with the NOS of < 6 points as low quality (16).

### Statistical Analysis

We assessed the consistency across the included studies using the Cochrane Q test and the I^2^ statistic. A *P* < 0.1 for the Q statistic or I^2^ ≥50% indicated substantial heterogeneity. We first collected the sample size and the number of events in the warfarin or DOAC groups and calculated their corresponding crude rates of effectiveness and safety outcomes. The comparison results between the warfarin or DOAC groups were expressed as HRs and 95%CIs. Second, we assessed the effectiveness and safety of DOACs vs. warfarin in patients with AF using the adjusted HRs. The adjusted HRs and 95%CIs were converted to the natural logarithms (Ln[HR]) and standard errors, which were pooled by a random-effects model using an inverse variance method.

All statistical analyses were conducted using the Review Manager Version 5.4 (the Nordic Cochrane Center, Rigshospitalet, Denmark). The statistical significance threshold was set at a *P* < 0.05.

## Results

The process of the literature retrieval is presented in Supplementary Figure 1. A total of 170 studies were identified through the electronic searches in the PubMed and Embase databases. According to the predefined criteria, we finally included 4 studies in this meta-analysis (14, 17–19). Table 1 shows the baseline patient characteristics of the included studies. All include studies are *hoc* RCT and the data sources are from effective anticoagulation with factor Xa next generation atrial fibrillation–thrombolysis in myocardial infarction 48 (ENGAGE AF-TIMI 48) (14), apixaban for reduction in stroke and other thromboembolic events in atrial fibrillation trial (ARISTOTLE TRIAL) (17), rivaroxaban once daily oral direct factor Xa inhibition compared with vitamin K antagonism for prevention of stroke and embolism trial in atrial fibrillation (ROCKET AF trial) (18), and randomized evaluation of long-term anticoagulant therapy (RE-LY) (19). Latin American includes Argentina Brazil, Chile, Colombia, Mexico, Peru, and Venezuela, and all the remaining countries included in the entire trial were considered to be non-LA countries. In total, 8,965 Latin American patients (5,096 taking DOACs and 3,869 taking warfarin) and 62,716 non-Latin American patients (37,315 taking DOACs and 25,401 taking warfarin) were included in this meta-analysis. All of these included studies had a moderate-to-high quality with the NOS score of ≥6 points.

**Table 1:** Clinical characteristics of the included studies.

	**Avezum-2018**		**Corbalán-2018**		**Bahit-2020**		**Blumer-2021**	
	**Latin** **American**	**Non-Latin American**	**Latin** **American**	**Non-Latin American**	**Latin American**	**Non-Latin American**	**Latin American**	**Non-Latin American**
Study design	*Post-hoc* analysis of RCT		*Post-hoc* analysis of RCT		*Post-hoc* analysis of RCT		*Post-hoc* analysis of RCT	
Date source	RE-LY		ENGAGE AF-TIMI 48		ARISTOTLE TRIAL		ROCKET AF trial	
DOACs	dabigatran		edoxaban		apixaban		rivaroxaban	
Efficacy outcomes	SSE Stroke Ischemic stroke Haemorrhagic stroke Myocardial infarction Death from any cause		SSE Stroke Ischemic stroke All cause death Cardiovascular death		SSE All cause death		SSE All cause death	
Safety outcomes	Life-threatening bleeding Total bleeding Major bleeding Intracranial hemorrhage Gastrointestinal bleeding Minor bleeding Any bleeding		Major bleeding Major or NMCR bleeding Intracranial hemorrhage Gastrointestinal bleeding, Any bleeding		Major bleeding Major or NMCR bleeding Intracranial hemorrhage		Major bleeding, Major or NMCR bleeding Intracranial hemorrhage	
Region	Argentina Brazil Colombia Mexico Peru	All remaining countries included in the entire trial were considered to be non-LA countries	Argentina Brazil Chile Colombia Guatemala Mexico Peru	NA	Argentina, Brazil Chile Colombia Puerto Rico Mexico	North America (USA, Canada) Europe (Austria, Belgium, Czech Republic, Denmark, Finland, France, Germany, Hungary, Israel, Italy, Netherlands, Norway, Poland, Romania, Spain, South Africa, Sweden, Turkey, United Kingdom, Ukraine) Asia Pacific (Australia, China, Hong Kong, India, Japan, Korea, Malaysia, Philippines, Singapore, Taiwan	Argentina Brazil Chile Colombia Mexico Peru Venezuela	rest of the world
Age (years)	71.6	71.5	71.4	70.5	71	69.7	75	72
Sex (% female)	-	-	40.6	30.3	38.6	34.5	42	39
No. of AF patients	956	17,157	2,661	18,444	3,486	14,733	1,878	12,386
BMI	-	-	-	-	29	-	27.8	28.3
**Pattern of atrial fibrillation (%)**								
Persistent	70.7	33.2	85.2	73	91.5	83.1	91	79
Paroxysmal	-	-	14.8	27	8.5	16.9	8	19
New onset/newly diagnosed	-	-	-	-	-	-	1	2
CHADS2 score	2.2	2.1	2.9	2.8	2.1	2.1	3.6	3.5
CHA2DS2-VASc score	3.5	3.6	4.2	4.3	-	-	3.6	3.5
**Comorbidities (%)**								
Prior stroke, TIA, or non-CNS embolism	11.5	12.6	29.8	28.1	13.8	17.1	56	55
Carotid or peripheral artery disease	-	-	-	-	-	-	7	9
Hypertension	82.3	78.7	95.2	93.4	89.1	87.1	93	90
Diabetes	-	-	28.5	37.2	-	-	39	40
Prior MI	-	-	6.4	12.3	9.8	15.2	11	18
CHF	41.1	31.5	63.4	56.6	38.3	34.8	60	63
COPD	-	-	-	-	-		7	11
**Medications (%)**								
Prior VKA use	44.0	63.0	48.0	60.5	45.8	42.1	61	63
Prior chronic aspirin use	48.4	39.1	-	-	33.0	30.4	38	36
ACE inhibitor/ARB	55.9	44.2			-	-	75	74
Beta-blocker	-	-	59.9	67.2	56.2	64.9	56	66
Renin, angiotensin, or aldosterone inhibitor	-	-	72.7	64.9	-	-	-	-
Calcium-channel blockers	-	-	18.4	33.0	-	-	-	-
Lipid lowering	-	-	28.3	50.6	-	-		
Diuretic agents	-	-	36.7	29	-	-	6.1	59
Digitalis	-	-	36.7	29	-	-	42	38
Amiodarone			19.5	10.7	-		14	7
Follow-up (year)	2.0		2.8		1.8		1.9	
Quality assessment	NOS = 9 points		NOS = 9 points		NOS = 9 points		NOS = 8 points	

*AF, atrial fibrillation; RCT, Randomized Controlled Trial; BMI, body mass index; EAST-AFNET 4, Early Treatment of Atrial Fibrillation for Stroke Prevention Trial; RE-LY, Randomized Evaluation of Long-Term Anticoagulant Therapy; ARISTOTLE TRIAL, Apixaban for reduction in stroke and other ThromboemboLic events in atrial fibrillation (ARISTOTLE) trial; ROCKET AF trial, (Rivaroxaban Once Daily Oral Direct Factor Xa Inhibition Compared with Vitamin K Antagonism for Prevention of Stroke and Embolism Trial in Atrial Fibrillation; SSE, stroke or systemic embolism; major or NMCR bleeding, major or non-major clinically relevant (NMCR) bleeding; CNS, central nervous system; BMI, body mass index; CHF, congestive Heart failure; MI, myocardial infarction; TIA, transient ischemic attack; VKA, vitamin K antagonist; COPD, chronic obstructive pulmonary disease; CHA2DS2-VASc, congestive heart failure/left ventricular ejection fraction ≤ 40%, hypertension, age ≥ 75 years (2 points), diabetes mellitus, prior stroke/transient ischemic attack/thromboembolism (2 points), vascular disease, age 65–74 years, female sex; ACEIs, angiotensin-converting enzyme inhibitors; ARBs, angiotensin receptor blockers; NOS, Newcastle-Ottawa Scale*.

### Crude Event Rates Between DOACs vs. Warfarin

In Latin American patients with AF, for the effectiveness outcomes shown in Supplementary Figure 2, the use of DOACs compared with warfarin was significantly associated with decreased risks of SSE [odds ratio (OR) = 0.79; 95% CI.64–0.99] and hemorrhagic stroke (OR = 0.13; 95% CI.05–0.33), but not stroke (OR = 0.76; 95% CI.54–1.07), ischemic stroke (OR = 1.19; 95% CI.80–1.78), all-cause death (OR = 0.91; 95%CI.78–1.07), and cardiovascular death (OR = 1.00; 95%CI.61–1.67). For the safety outcomes in Supplementary Figure 3, compared with warfarin, the use of DOACs was associated with reduced risks of major or NMCR bleeding (OR = 0.72; 95%CI.56–0.94), major bleeding (OR = 0.72; 95% CI.53–0.98), ICH (OR = 0.43; 95% CI.21–0.88), and any bleeding (OR = 0.66; 95% CI.57–0.78), but not gastrointestinal bleeding (OR = 0.65; 95% CI.10–3.99).

For patients treated with anticoagulants in non-Latin American patients with AF, for the effectiveness outcomes in Supplementary Figure 4, the use of DOACs compared with warfarin use was significantly associated with decreased risks of SSE (OR = 0.87; 95% CI.76–1.00), hemorrhagic stroke (OR = 0.41; 95% CI.25–0.67), all-cause death (OR = 0.89; 95% CI.84–0.95), cardiovascular death (OR = 0.87; 95% CI.79–0.95), but not stroke (OR = 0.92; 95%CI.69–1.22), ischemic stroke (OR = 1.08; 95% CI.82–1.42). For the safety outcomes in Supplementary Figure 5, compared with warfarin use, the use of DOACs was associated with reduced risks of major bleeding (OR = 0.77; 95% CI.62–0.95), ICH (OR = 0.43; 95%CI.35–0.53), and any bleeding (OR = 0.62; 95% CI.43–0.88), but not major or NMCR bleeding (OR = 0.80; 95%CI.61–1.04) and gastrointestinal bleeding (OR = 0.90; 95%CI.78–1.04).

### Adjusted Data of Outcomes Between DOACs vs. Warfarin

**Figure 1:**
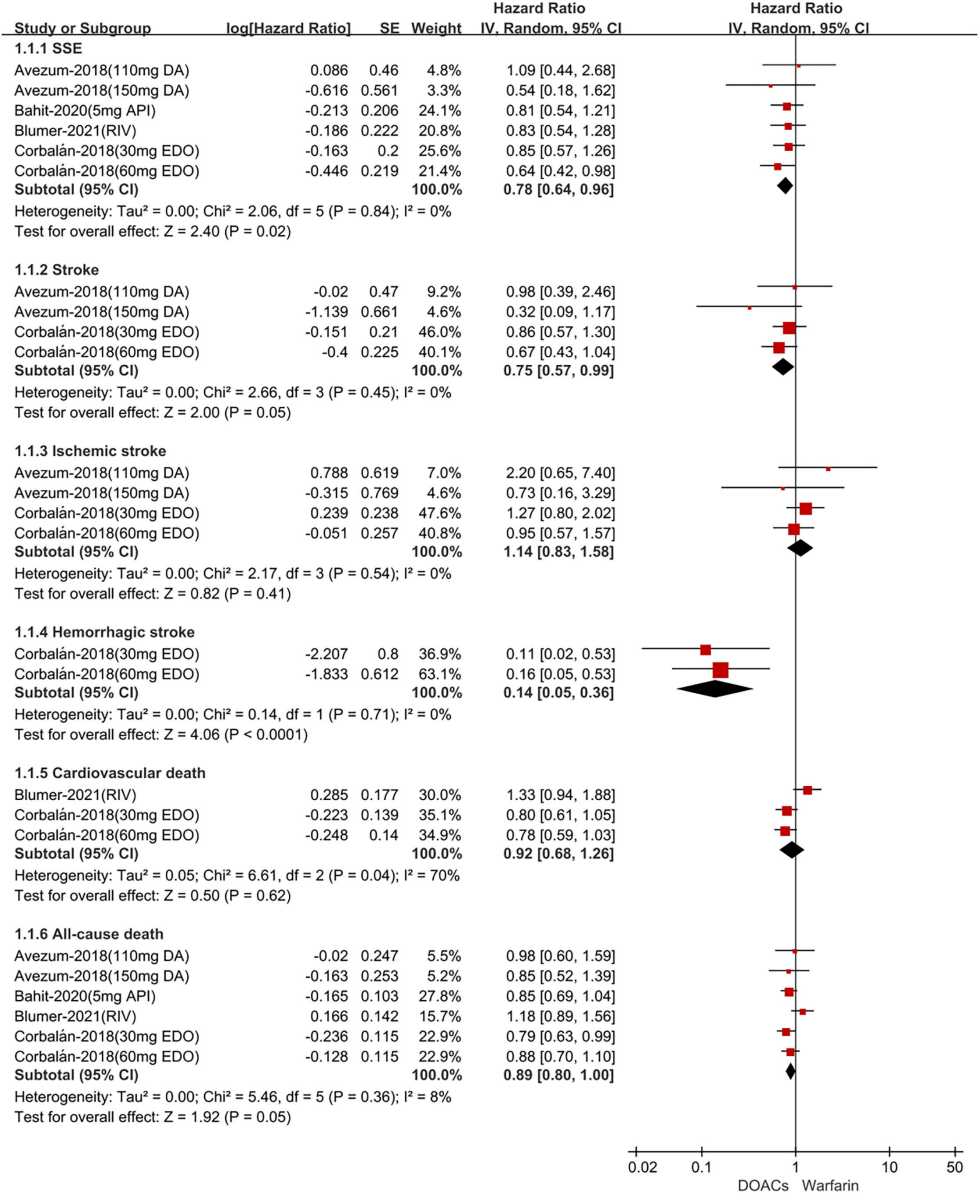
Adjusted effectiveness date of direct oral anticoagulants compared with warfarin in Latin patients with atrial fibrillation. DOACs, direct oral anticoagulants; DA, dabigatran; API, apixaban; EDO, edoxaban; RIV, rivaroxaban; SSE, stroke or systemic embolism; CI, confidence interval.

**Figure 2:**
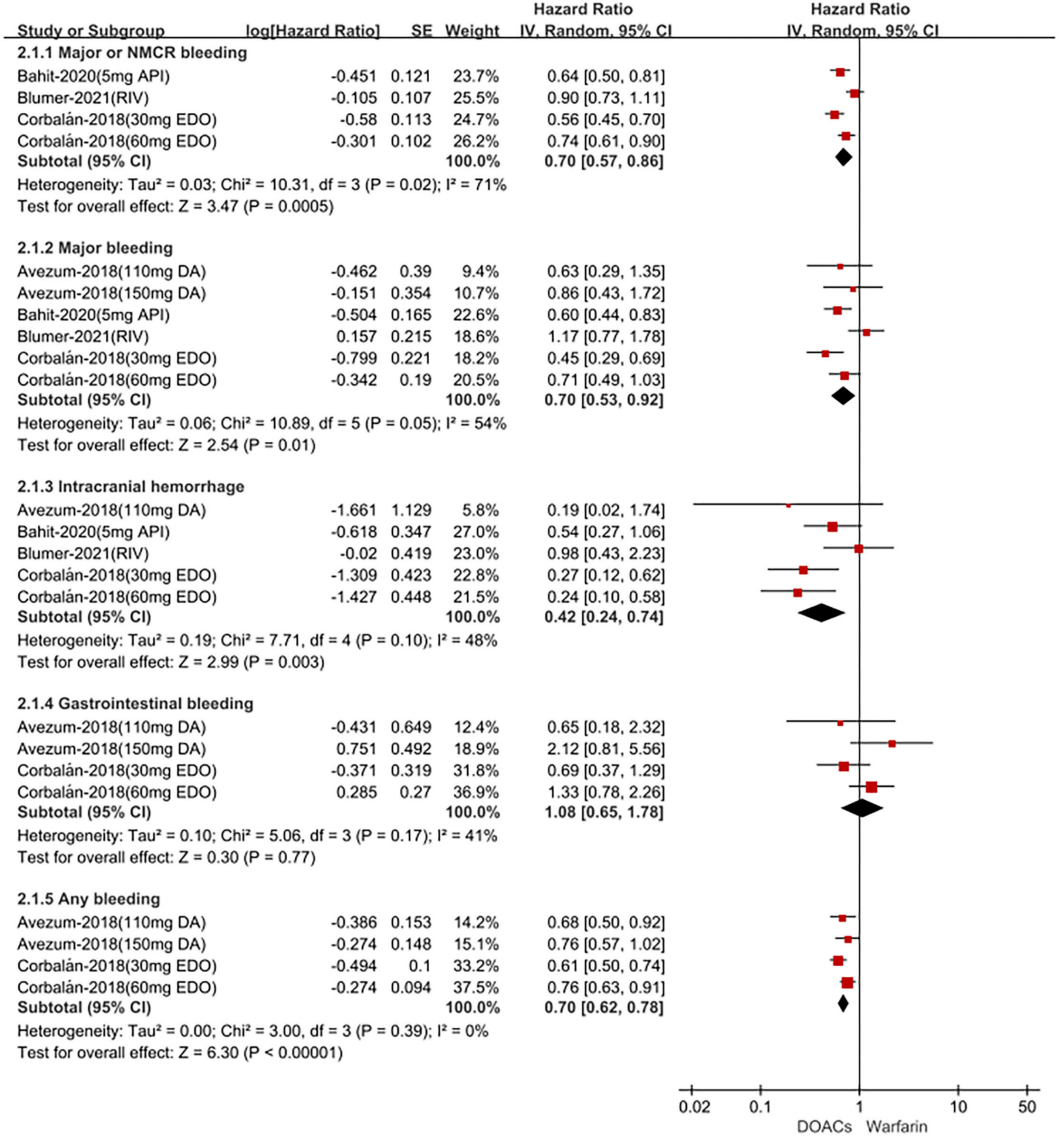
Adjusted safety date of direct oral anticoagulants compared with warfarin in Latin patients with atrial fibrillation. DOACs, direct oral anticoagulants; DA, dabigatran; API, apixaban; EDO, edoxaban; RIV, rivaroxaban; CI, confidence interval.

In Latin American patients with AF, for the effectiveness outcomes shown in Figure 1, the use of DOACs compared with warfarin was significantly associated with decreased risks of SSE (HR = 0.78; 95% CI.64–0.96), stroke (HR = 0.75; 95%CI.57–0.99), hemorrhagic stroke (HR = 0.14;95%CI.05–0.36), all-cause death (HR = 0.89; 95%CI.80–1.00), but not ischemic stroke (HR = 1.14; 95%CI.83–1.58) and cardiovascular death (HR = 0.92; 95%CI.68–1.26). For the safety outcomes in Figure 2, compared with warfarin, the use of DOACs was associated with reduced risks of major or NMCR bleeding (HR = 0.70; 95%CI.57–0.86), major bleeding (HR = 0.70; 95%CI.53–0.92), ICH (HR = 0.42; 95% CI.24–0.74), and any bleeding (HR = 0.70; 95%CI.62–0.78), but not gastrointestinal bleeding (HR = 1.08; 95% CI.65–1.78).

**Figure 3:**
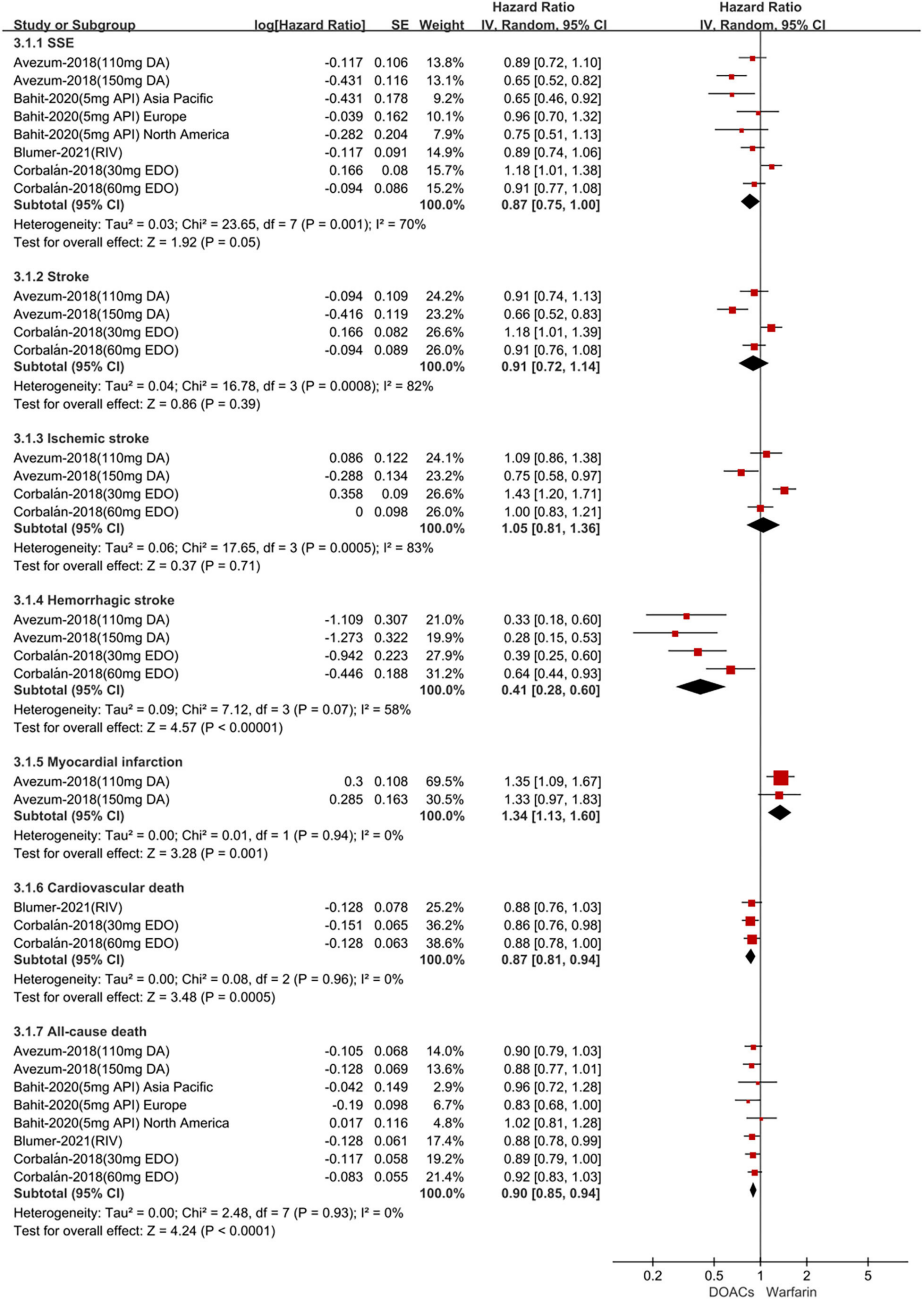
Adjusted effectiveness date of direct oral anticoagulants compared with warfarin in non-Latin patients with atrial fibrillation. DOACs, direct oral anticoagulants; DA, dabigatran; API, apixaban; EDO, edoxaban; RIV, rivaroxaban; SSE, stroke or systemic embolism; CI, confidence interval.

**Figure 4:**
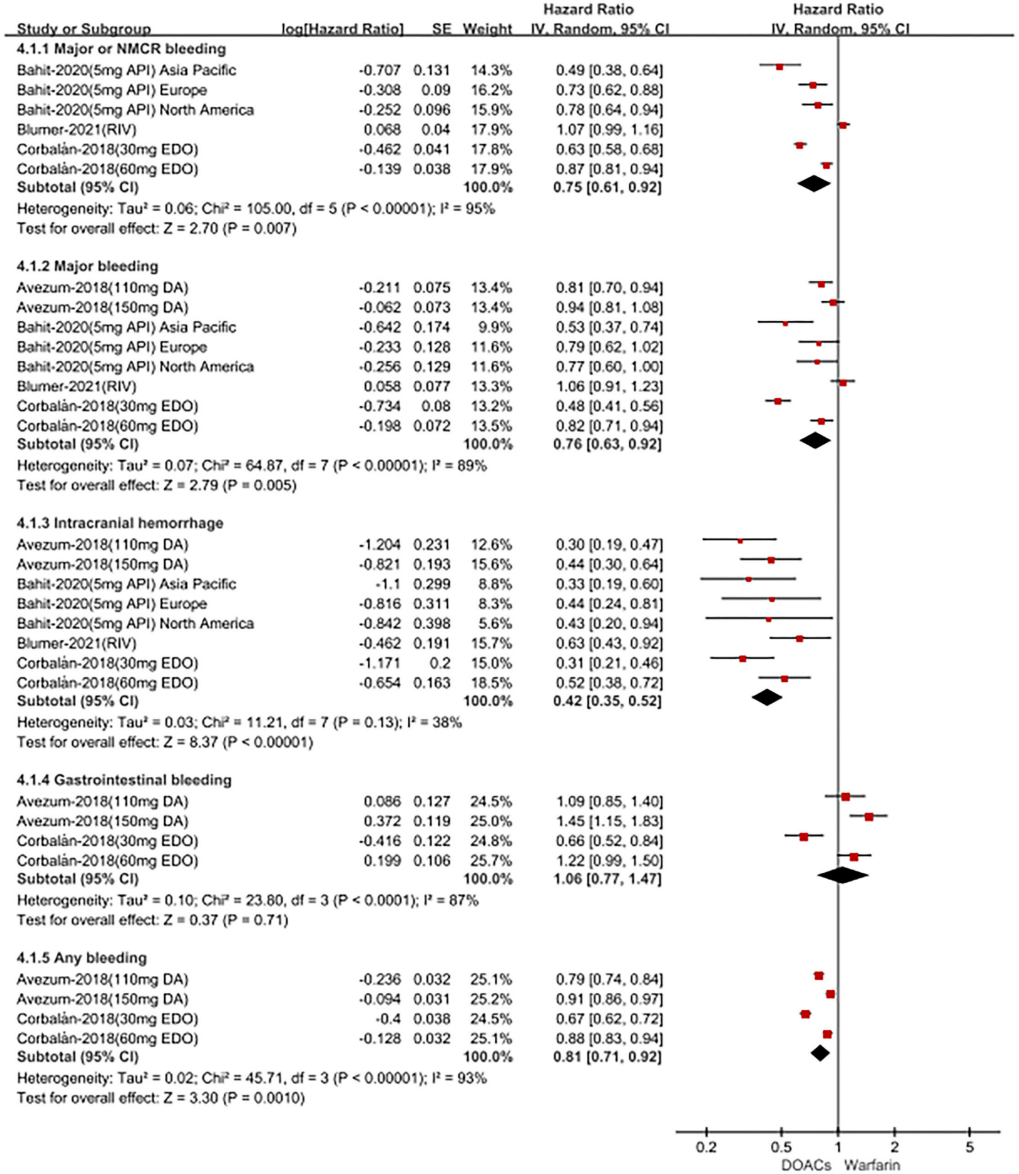
Adjusted safety date of direct oral anticoagulants compared with warfarin in non-Latin patients with atrial fibrillation. DOACs, direct oral anticoagulants; DA, dabigatran; API, apixaban; EDO, edoxaban; RIV, rivaroxaban; CI, confidence interval.

**Figure 5:**
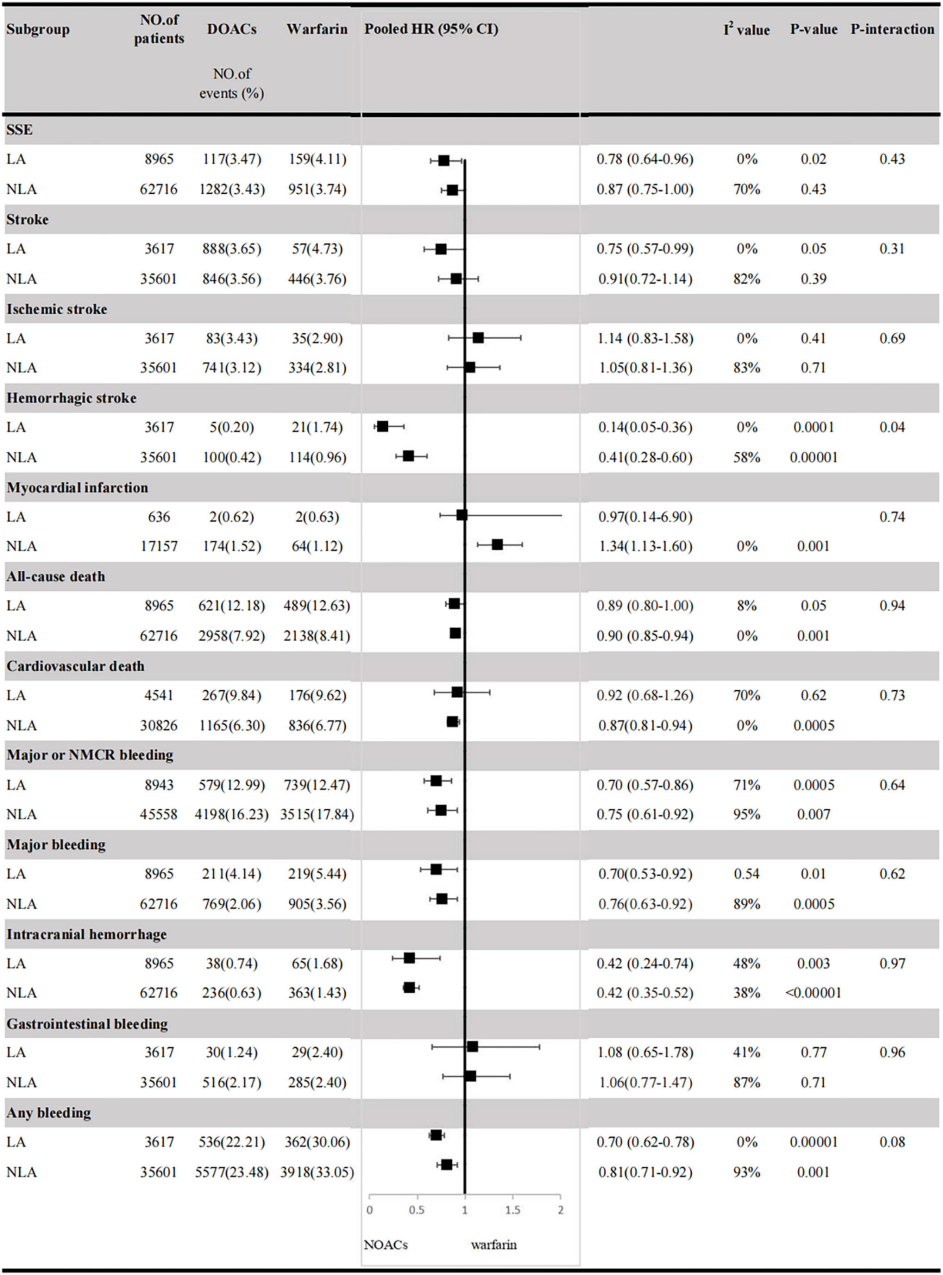
Efficacy and safety outcomes in AF patients from Latin American and non-Latin American. SSE, stroke or systemic embolism; CI, confidence interval; HR, hazard ratio; LA, Latin American; NLA, non-Latin American; CI, confidence interval; major or NMCR bleeding, major or non-major clinically relevant bleeding.

For patients treated with anticoagulants in non-Latin American patients with AF, for the effectiveness outcomes in Figure 3, the use of DOACs compared with warfarin was significantly associated with decreased risks of SSE (HR = 0.87; 95%CI.75–1.00), hemorrhagic stroke (HR = 0.41; 95%CI.28–0.60), cardiovascular death (HR = 0.87; 95% CI.81–0.94), all-cause death (HR = 0.90; 95%CI.85–0.94), conversely, increasing the risk of myocardial infarction (HR = 1.34; 95%CI 1.13–1.60), but not stroke (HR = 0.91; 95%CI.72–1.14) and ischemic stroke (HR = 1.05; 95% CI.81–1.36). For the safety outcomes in Figure 4, compared with warfarin use, the use of DOACs was associated with reduced risks of major or NMCR bleeding (HR = 0.75; 95% CI.61–0.92), major bleeding (HR = 0.76; 95%CI.63–0.92), ICH (HR = 0.42; 95% CI.36–0.52) and any bleeding (HR = 0.81; 95%CI.71–0.92), but not gastrointestinal bleeding (HR = 1.06; 95%CI.77–1.47). Not only that, we also conducted a summary analysis of the adjusted data of outcomes between Latin American patients and non-Latin American patients in Figure 5. The *P*-interaction between Latin American patients and non-Latin American patients with AF was no significant difference.

### Publication Bias

We have not performed an analysis of publication bias due to only 4 studies were included in our meta-analysis. It was noted that the publication bias should not be evaluated for some reported outcomes when fewer than 10 studies were included.

## Discussion

The main findings of our study were as follows: (1) DOAC use resulted in lower rates of SSE, stroke, hemorrhagic stroke, all-cause death, and associated with safer profiles (lower major or NMCR bleeding, major bleeding, ICH, and any bleeding) than warfarin in Latin American patients with AF; (2) DOAC use resulted in lower rates of SSE, hemorrhagic stroke, all-cause death, cardiovascular death, and associated with safer profiles (lower major or NMCR bleeding, major bleeding, ICH, and any bleeding) than warfarin in non-Latin American patients with AF; (3) DOAC use increased the risk of myocardial infarction than warfarin in non-Latin American patients with AF, but not in Latin American patients with AF; (4) in comparison to VKAs, DOACs were non-inferior regarding the outcomes of ischemic stroke, cardiovascular death, and gastrointestinal bleeding in Latin American patients with AF and the outcomes of stroke, ischemic stroke, and gastrointestinal bleeding in non- Latin American patients.

Important differences in clinical characteristics, response to treatment, and outcomes of patients with AF exist in the diverse regions of the world. Previous studies have shown that Latin American patients with AF are suffering from higher risks of death and embolism than non-Latin American patients with AF (20, 21). Actually, there are many reasons for the increased risk of death and embolism in Latin American patients with AF. Life expectancy differed substantially across cities within the same country. Cause-specific mortality also varied across cities, with some causes of death (unintentional and violent injuries and deaths) showing large variation within countries, whereas other causes of death (communicable, maternal, neonatal and nutritional, cancer, cardiovascular disease, and other non-communicable diseases) varied substantially between countries. These results highlight considerable heterogeneity in life expectancy and causes of death across cities of Latin America (22). Moreover, heterogeneity of risk factors (23–25) and socioeconomic conditions, public awareness, and availability of healthcare services that influence outcomes of diseases differ substantially between countries (26, 27) in Latin America and still need to be taken into account. Furthermore, inadequate prescription for medications associated with death reduction might also affect the prognosis of Latin American patients with AF (14). Therefore, antithrombotic therapy is particularly important to reduce the risk of embolism in Latin American patients with AF. Previous meta-analyses including the *post-hoc* analyses and sub-analyses of DOAC RCTs showed that there is a non-inferiority of DOACs compared with warfarin in Latin American patients with AF (15). Compared to the previous study, the RCTs included in this meta-analysis are outdated. More importantly, the number of available clinical studies are small and the results are controversial. In recent years, several new *post-hoc* analyses of RCTs not only examined the association between region and efficacy and safety outcomes but also explored the use of individual DOACs compared with warfarin in Latin American patients. The RCTs provide more endpoint events and arrive at different conclusions. Therefore, we aimed to reassess the effectiveness and safety outcomes of DOACs vs. warfarin in Latin American and non-Latin American patients with AF. Our meta-analysis shows that DOACs appeared to have significant reductions in SSE, stroke, hemorrhagic stroke, all-cause death, major or NMCR bleeding, major bleeding, ICH, and any bleeding, but showed comparable rates of ischemic stroke, cardiovascular death, and gastrointestinal bleeding in Latin American patients with AF. DOACs appeared to have significant reductions in SSE, hemorrhagic stroke, all-cause death, cardiovascular death, major or NMCR bleeding, major bleeding, ICH, and any bleeding and increased the risk of myocardial infarction, but comparable risks of stroke, ischemic stroke, and gastrointestinal bleeding in non-Latin American patients with AF. In addition, we assessed crude event rates of outcomes between DOACs vs. warfarin in Latin/non-Latin American patients with AF. Overall, in comparison to warfarin, DOACs had lower or similar rates of thromboembolic and bleeding risk, which was consistent with a previous study (15). Interestingly, we found that DOACs increased the risk of myocardial infarction compared with warfarin in non-Latin American patients with AF. The result was derived from the RE-LY study, which included patients using dabigatran. Previous studies have warned this risk (28, 29). Prospective data on dabigatran in this population undergoing PCI are still needed.

It is worth pointing out that DOACs have advantages over warfarin such as short onset time, short half-life, low inter- and intra-individual variability, and drug-drug interactions. The current international guidelines recommend the use of DOACs as replacement therapy for VKAs in patients with non-valvular AF because it has more effective, safer, and more convenient features. Different from DOACs, the anticoagulant activity of VKAs depends on TTR (time in therapeutic range). Among the included studies, the mean TTR of VKAs users in Latin America ranged from 58 to 66%, which was not higher than that of non-Latin Americans overall and lower than what is recommended in the guidelines (1, 30). Therefore, DOACs may be regarded as a safer alternative to VKAs in Latin American patients with AF. Although no observational studies have been carried out to directly compare the use of DOACs and warfarin in Latin American patients with AF, several studies have validated the benefits of the use of DOACs in this population. Data from the GLORIA-AF (Global Registry on Long-Term Antithrombotic Treatment in Patients with Atrial Fibrillation, Phase II) study indicated the consistent safety and effectiveness of dabigatran in Latin American patients with AF during a 2-years follow-up (31). Moreover, the XANTUS-EL (Xarelto for Prevention of Stroke in Patients With Atrial Fibrillation in Eastern Europe, the Middle East and Africa [EEMEA], and Latin America) study confirmed the benefits of rivaroxaban for stroke prevention in patients with non-valvular AF from Eastern Europe, the Middle East, Africa, and Latin America (32). However, the results concerning whether DOACs are more cost-effective than warfarin in Latin America remain a controversy (33). The evidence provided by our meta-analysis may offer some confidence to clinicians when selecting DOACs for Latin American patients who need anticoagulation therapy, especially for those at a high risk of bleeding. The present results support that the use of DOACs is at least non-inferior to warfarin in Latin American patients with AF and provides an effective anticoagulant choice without monitoring. Further studies should be performed to clarify this problem.

### Limitations

Several limitations should be acknowledged. First, because of the small number of included studies, we did not perform subgroup analysis based on dosage or type of DOACs. Second, individual patient-level data from trials were not available, and some of the patients in Latin American countries enrolled might not be ethnically Latin American. Third, the results of the present analysis do not represent all countries in Latin America, as a limited number of countries in this region were included. Finally, we cannot exclude the possibility that there is potential confounding or interaction between enrollment in Latin America and anticoagulants.

## Conclusion

The current pooled data from the four *post-hoc* analyses of RCTs suggested that compared with warfarin, DOACs appeared to have significant reductions in SSE, stroke, hemorrhagic stroke, all-cause death, major or NMCR bleeding, major bleeding, ICH, and any bleeding, but comparable risks of ischemic stroke, cardiovascular death, and gastrointestinal bleeding in Latin American patients with AF. DOACs appeared to have significant reductions in SSE, hemorrhagic stroke, all-cause death, cardiovascular death, major or NMCR bleeding, major bleeding, ICH, and any bleeding, and increased the risk of myocardial infarction, but comparable risks of stroke, ischemic stroke, and gastrointestinal bleeding in non-Latin American patients with AF.

## Data Availability Statement

The original contributions presented in the study are included in the article/Supplementary Material, further inquiries can be directed to the corresponding authors.

## Author Contributions

All authors listed have made a substantial, direct, and intellectual contribution to the work and approved it for publication.

## Conflict of Interest

The authors declare that the research was conducted in the absence of any commercial or financial relationships that could be construed as a potential conflict of interest.

## Publisher's Note

All claims expressed in this article are solely those of the authors and do not necessarily represent those of their affiliated organizations, or those of the publisher, the editors and the reviewers. Any product that may be evaluated in this article, or claim that may be made by its manufacturer, is not guaranteed or endorsed by the publisher.
