# Supplementary material for: Direct Oral Anticoagulants vs. Warfarin in Latin American Patients With Atrial Fibrillation: Evidence From Four post-hoc Analyses of Randomized Clinical Trials
Source: Front Cardiovasc Med. 2022 Mar 4;9:841341. doi: 10.3389/fcvm.2022.841341 (PMC8930927; doi:10.3389/fcvm.2022.841341)
Supplement: Supplementary file 1 [file Data_Sheet_1.docx]

**Supplementary Table 1. The search strategies of this meta-analysis**

| Databases | Keywords | Results |
| --- | --- | --- |
| PubMed |  |  |
| #1 | 'atrial fibrillation' OR 'non-valvular atrial fibrillation' OR 'atrial flutter' | 106874 |
| #2 | 'Latin American' OR 'Latin ' | 37977 |
| #3 | 'non-vitamin K antagonist oral anticoagulants' OR 'NOACs' OR 'new oral anticoagulants' OR 'novel oral anticoagulants' OR 'direct oral anticoagulants'  OR 'DOACs' OR 'oral thrombin inhibitors' OR 'oral factor Xa inhibitors'  OR 'dabigatran' OR 'rivaroxaban' OR 'apixaban' OR 'edoxaban' | 16627 |
| #4 | 'vitamin k antagonists' OR 'warfarin' | 34954 |
| #5 | #1 and #2 and #3 and #4 | 19 |
| EMBASE |  |  |
| #1 | 'atrial fibrillation' OR 'non-valvular atrial fibrillation' OR 'atrial flutter' | 211818 |
| #2 | 'South and central America' OR 'Latin American' OR 'Latin' | 284914 |
| #3 | 'non-vitamin K antagonist oral anticoagulants' OR 'NOACs' OR 'new oral anticoagulants' OR 'novel oral anticoagulants' OR 'direct oral anticoagulants'  OR 'DOACs' OR 'oral thrombin inhibitors' OR 'oral factor Xa inhibitors'  OR 'dabigatran' OR 'rivaroxaban' OR 'apixaban' OR 'edoxaban' | 709237 |
| #4 | 'vitamin k antagonists' OR 'warfarin' | 101975 |
| #5 | #1 and #2 and #3 and #4 | 147 |


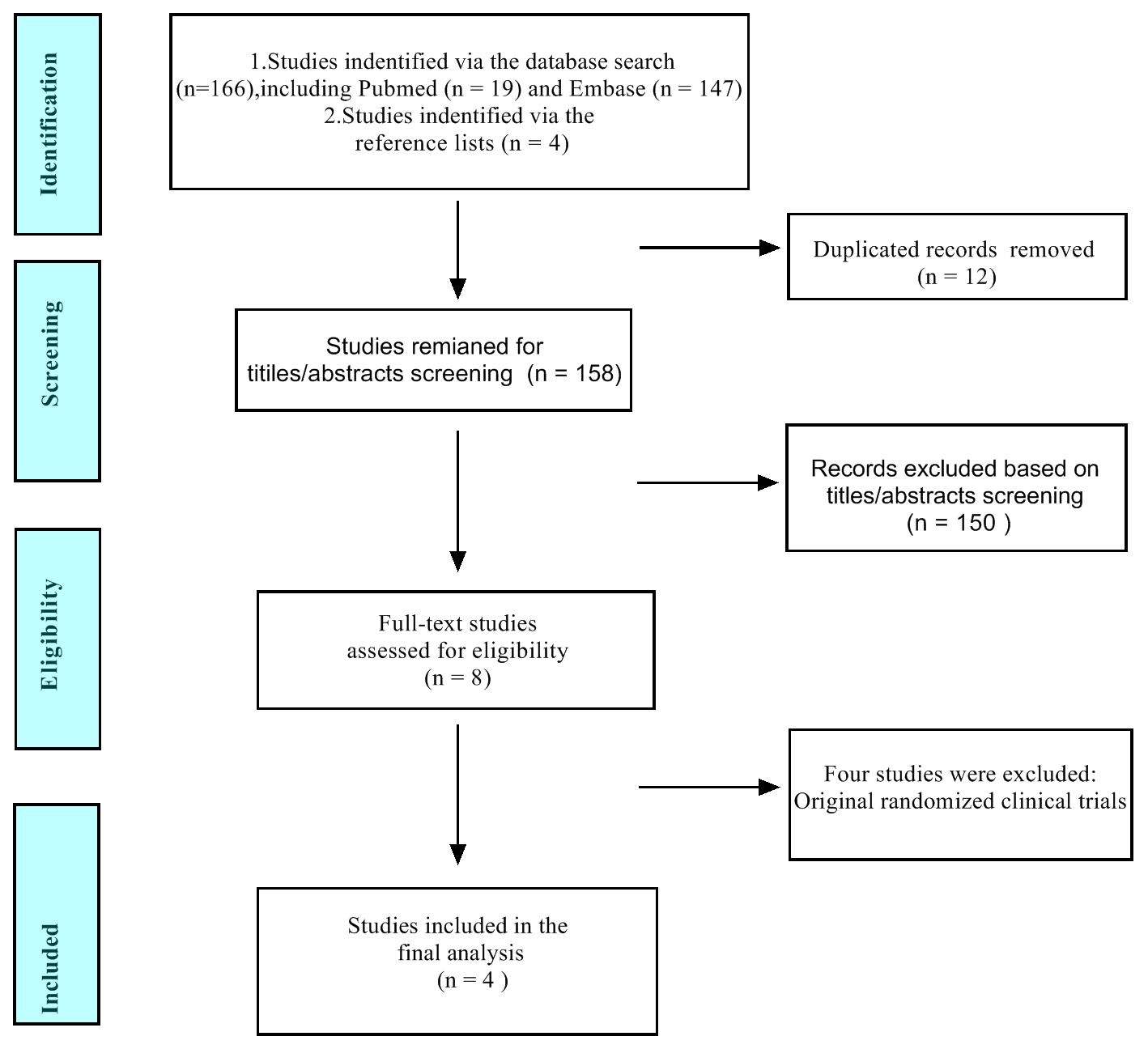


**Supplementary Figure 1. The process of the literature retrieval of this meta-analysis**
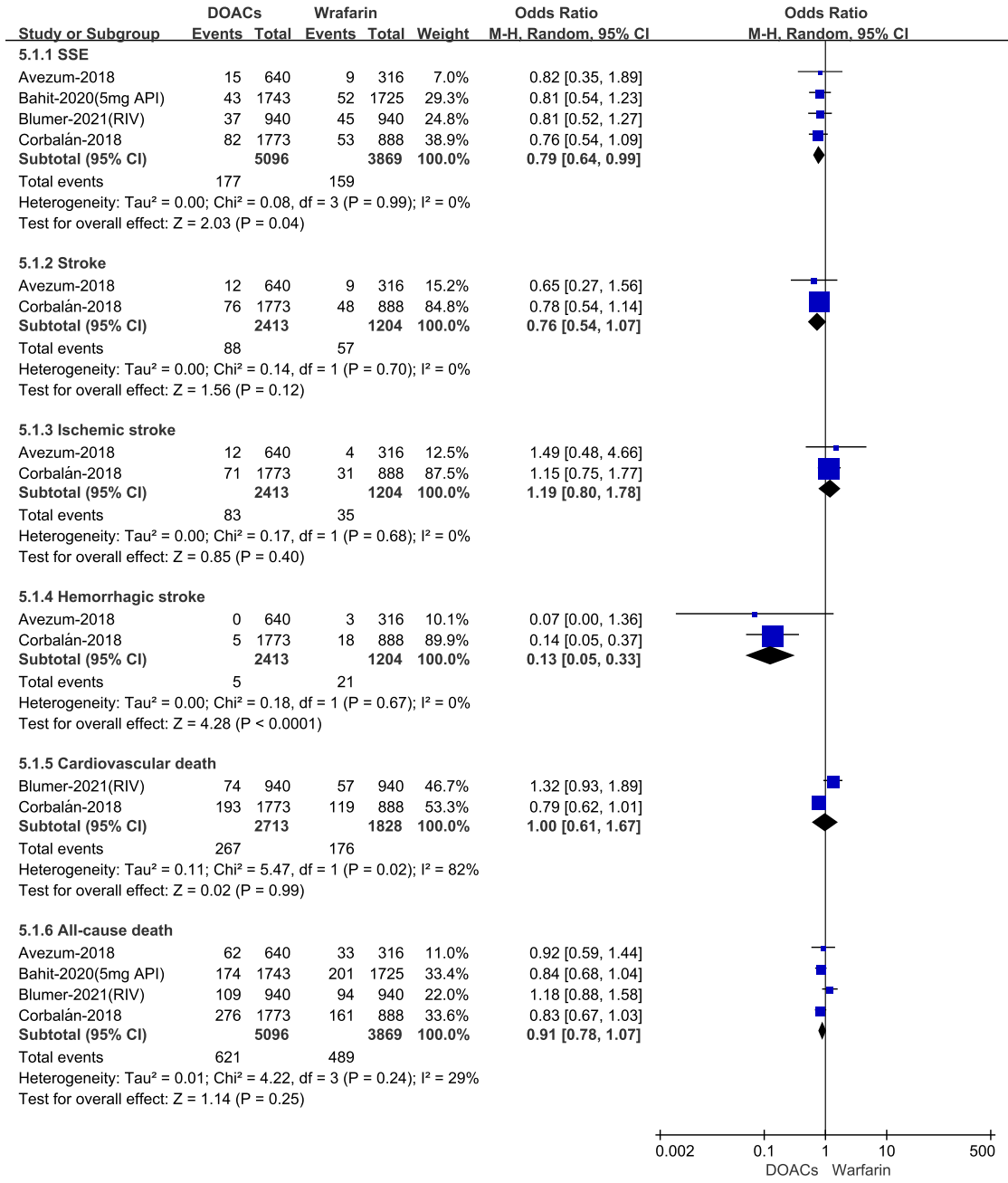


**Supplementary Figure 2:** Crude effectiveness event rates of direct oral anticoagulants compared with warfarin in Latin patients with atrial fibrillation.

Abbreviation: DOACs = direct oral anticoagulants ; DA = dabigatran ; API = apixaban; EDO = edoxaban; RIV = rivaroxaban; SSE=stroke or systemic embolism;CI = confidence interval ;
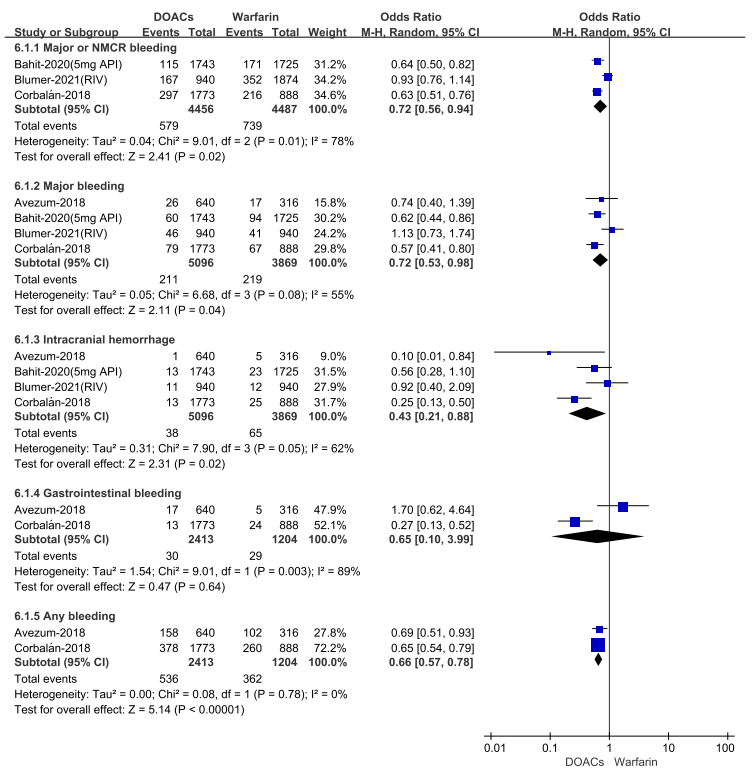


**Supplementary Figure 3:** Crude safety event rates of direct oral anticoagulants compared with warfarin in Latin patients with atrial fibrillation.

Abbreviation: DOACs = direct oral anticoagulants ; DA = dabigatran ; API = apixaban; EDO = edoxaban; RIV = rivaroxaban; CI = confidence interval ; major or NMCR bleeding=major or non-major clinically relevant (NMCR) bleeding


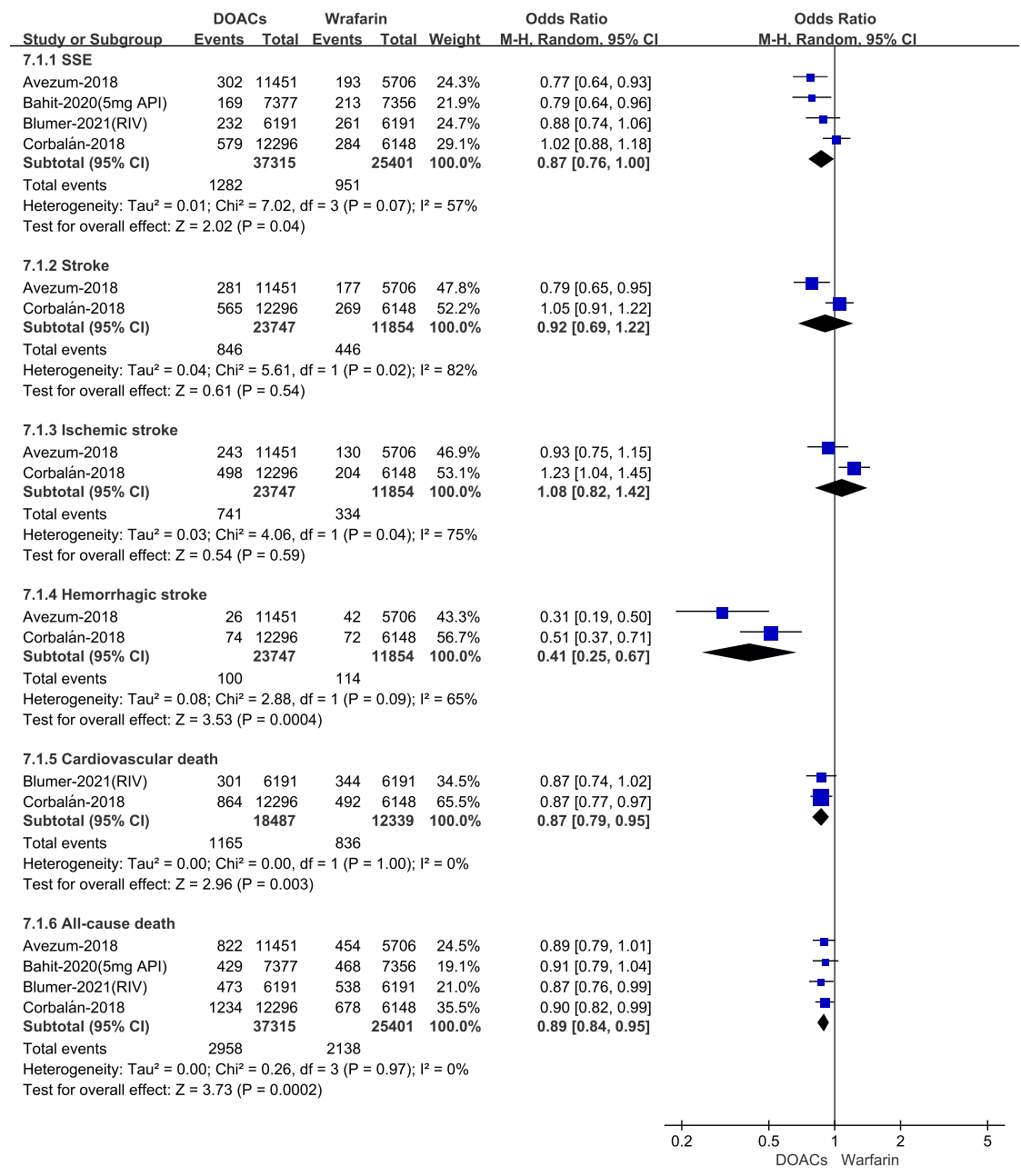


**Supplementary Figure 4:** Crude effectiveness event rates of direct oral anticoagulants compared with warfarin in non-Latin patients with atrial fibrillation.

Abbreviation: DOACs = direct oral anticoagulants ; DA = dabigatran ; API = apixaban; EDO = edoxaban; RIV = rivaroxaban; SSE=stroke or systemic embolism;CI = confidence interval ;


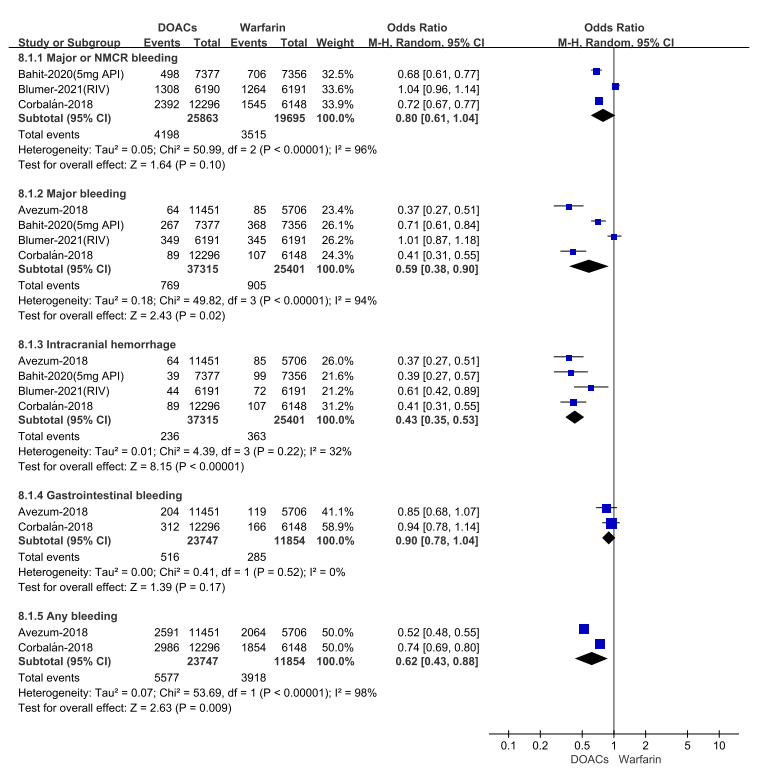


**Supplementary Figure 5:** Crude safety event rates of direct oral anticoagulants compared with warfarin in non-Latin patients with atrial fibrillation.

Abbreviation: DOACs = direct oral anticoagulants ; DA = dabigatran ; API = apixaban; EDO = edoxaban; RIV = rivaroxaban; CI = confidence interval ; major or NMCR bleeding=major or non-major clinically relevant (NMCR) bleeding
